# The Complete Genome Sequences of Bacteriophages ASegato, DejaVu, Judebell, and RicoCaldo isolated using *Microbacterium foliorum*

**DOI:** 10.17912/micropub.biology.001443

**Published:** 2025-02-19

**Authors:** Robert Logan, Mahlet Abebe Biratu, Maria Andreea Busila, Ignacio Fernandez Busto, Nathan Caldwell, Peyton Chestnut, Hycell Colmenares Duno, Ricardo Cuello, Dhazyah Johnson, Jeniush Kark, Curvan Lawrnece, Jhemerial Arnique Lewis, Wod-Dardely Maglorie, Andrea Mendoza, Wesely Mills, Edoardo Miranda Colombo, Isabela Pacifico, Olivia Peters, Helen Pham, Izabel Renee Pozar, Madison Rearick, Jenna Reed, Ana Romero, Beatriz De Oliveira Segato, Hayden Turner, Sami Walaieh, Matthew Waterman

**Affiliations:** 1 Life and Chemical Sciences, Eastern Nazarene College, Quincy, Massachusetts, United States; 2 Biology and Biotechnology, Endicott College, Beverly Cove, Massachusetts, United States

## Abstract

We report the discovery and characterization of bacteriophages ASegato, DejaVu, Judebell, and RicoCaldo, isolated from grass samples collected in Quincy, Massachusetts, using *Microbacterium foliorum* B-24224 as the isolation host. Based on gene content similarity, these phages are assigned to actinobacteriophage clusters ED2, ED1, EG, and EK2 respectively.

**Figure 1. Representative negative-staining (1 % uranyl acetate) transmission electron micrographs of the phages:**
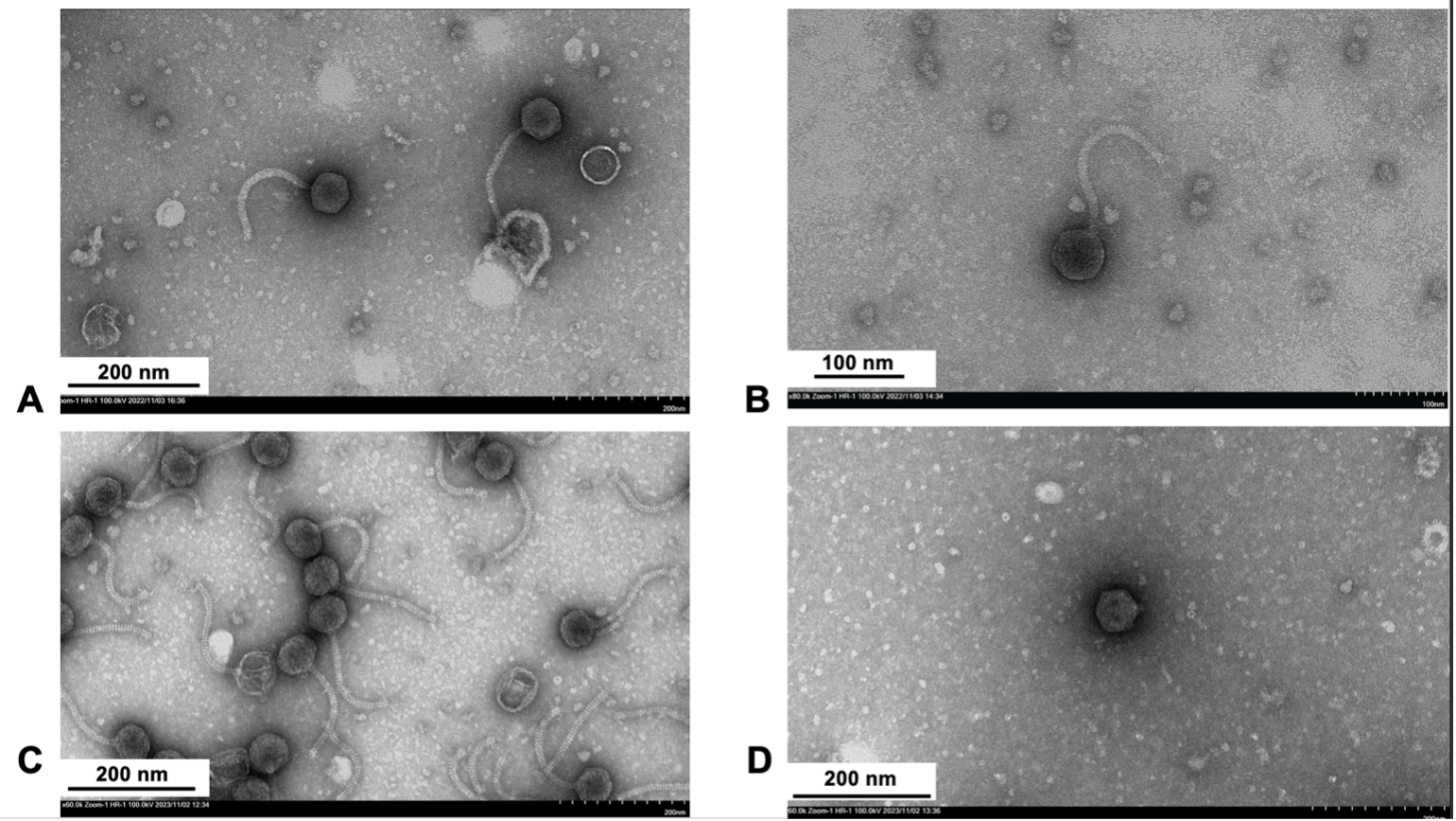
A) ASegato, B) DejaVu, C) Judebell, and D) RicoCaldo, with capsid diameters of ~ 68 nm, 60 nm, 57 nm, and 57 nm, respectively.

## Description

Microbacterium spp. are Gram-positive bacteria often found on plants, in soil, water, and dairy products, and have also been reported in clinical specimens (Gneiding et al., 2008) . As an avenue to control growth of *Microbacterium* , either for phage therapy or to minimize contaminations during food production, the isolation and characterization of bacteriophages capable of infecting bacteria of this genus is important. Here, we present the isolation and characterization of four bacteriophages using *Microbacterium foliorum* B-24224.

Bacteriophages ASegato, DejaVu, Judebell, and RicoCaldo were isolated from grass samples collected in Quincy, Massachusetts, US (Table 1). Each of the grass samples were collected in separate zipper plastic storage bags. Bacteriophages were then collected by first agitating the sample for 1 minute in 20 mL of peptone-yeast extract-calcium (PYCa) liquid medium. Then, 3 mL of the wash was withdrawn using a 5 mL syringe and passed through a sterile Whatman GD/X 0.2 μM PES filter. 500 μL of the filtered wash was then mixed with 500 μL of a saturated culture of Microbacterium foliorum and allowed to sit undisturbed for 10 minutes before 3 mL of warm molten PYCa top agar was added to the mix and immediately plated on PYCa agar plates. The plates were incubated for 24 hr at 30 ˚C, yielding plaques of ASegato, DejaVu, Judebell, and RicoCaldo, which were then purified through 3 additional rounds of plating (Table 1). Negative stain (1% uranyl acetate) transmission electron microscopy revealed ASegato, DejaVu, and Judebell to have a siphovirus morphology while RicoCaldo has a podovirus morphology ( Figure 1 ).

DNA was extracted from bacteriophage lysates using the Wizard DNA Clean-Up System (Promega). Subsequently, the genomes were sequenced with an Illumina MiSeq sequencer (v3 reagents) following library preparation using NEB FS Ultra II kit. Raw reads were then assembled using Newbler v2.9 and assessed for completeness and genome termini using Consed v29, both with default parameters (Gordon et al., 1998, Margulies et al., 2005) . Sequencing data and genome characteristics are provided in Table 1.

All genomes were automatically annotated using Glimmer v3.02 and GeneMarkS v3.28 (Besemer et al., 2001, Besemer et al., 2005, Delcher et al., 2007) . Manual refinement of the annotation made use of Phamerator Actino_draft v578, DNA Master v5.23.6 (http://cobamide2.bio.pitt.edu), and PECAAN v20221109 (Pope et al., 2017, Rinehart et al., 2016, Pope et al., 2017) . Genes that encode for tRNA were predicted using Aragorn v1.2.38 and tRNAscanSE v2.0 (Laslett et al., 2004, Lowe et al., 2016) . Transmembrane proteins were detected by using TMHMM and TOPCONS (Krogh et al., 2001, Tsirigos et al., 2015) . Prediction of protein function was accomplished using HHPred searches against the PDB mmCIF70 8Mar, Pfam-A v37, UniProt-SwissProt-viral70 3, and NCBI Conserved Domainsv3.19 databases and BLASTp searches against the NCBI non-redundant and Actinobacteriophage databases (Altschul et al., 1997, Söding et al., 2005) . Based on gene content similarity of at least 35% to phages in the Actinobacteriophage database, phagesdb (https://phageDB.org), ASegato and DejaVu are assigned to cluster ED (ED2 and ED1 subclusters, respectively), Judebell to cluster EG cluster, and RicoCaldo to subcluster EK2 subcluster (Table 1.) (Pope et al., 2017 Russell and Hatfull 2017p) . All software were used with default settings.

As characteristic of phages in the ED, EG, and EK clusters, none of these newly isolated phages encode identifiable immunity repressor or integrase functions, suggesting they are unlikely to establish lysogeny. RicoCaldo, as is typical for EK phages, encodes a large gene of unknown functions that is 13,470 bp long and which spans 25% of its genome length (Jacobs et al., 2020) .

Data Availability

Genomes are available at GenBank with the following accession numbers: ASegato (OR521059.1), DejaVu (OR475263.1), Judebell (PP946909.1) and RicoCaldo (PP946910.1). Sequencing reads for these genomes are available through the NCBI SRA database with the following SRA accession numbers: ASegato (SRX23452925), DejaVu (SRX23452931), Judebell (SRX26476175), and RicoCaldo (SRX26476176).

Acknowledgements

We are grateful for the remarkable support of Daniel Russell, Rebecca Garlena, Viknesh Sivanathan, William Biederman, Debra Jacobs-Sera, the Hatfull lab, and the entire leadership within the HHMI SEA-PHAGES program. Their support has included efforts in DNA sequencing, genome assembly, reviewing manuscripts and annotations, providing education for faculty and students, and in administrative work.

Table 1 Isolation and sequencing parameters, and plaque and genome characteristics

**Table:** 

Phage	ASegato	DejaVu	Judebell	RicoCaldo
Sample location GPS coordinates	42.27307 N, 71.01037 W	42.2703 N, 71.01062 W	42.27195 N, 71.01153 W	42.271501 N, 71.011677 W
Plaque morphology	Halo with clear center	Halo with clear center	Halo with clear center	Clear
150-base sequencing reads #	321,892	176,383	309,122	391,445
Approximate shotgun coverage	754	415	715	1028
Genome length (bp)	63,246	63,339	61,786	54,524
Genome termini	Direct terminal repeat of 3797 bp	Direct terminal repeat of 2959 bp	Direct terminal repeat of 204 bp	Circularly permuted
% GC	61.7	62.8	67	59.7
# ORFs (# with predicted function)	121 (26)	123 (25)	105 (28)	52 (14)
# tRNAs	3	0	0	0
Cluster ID	ED2	ED1	EG	EK2
