# Corrigendum: The Complete Genome Sequences of Bacteriophages ASegato, DejaVu, Judebell, and RicoCaldo isolated using *Microbacterium foliorum*

**DOI:** 10.17912/micropub.biology.001617

**Published:** 2025-04-28

## Description

For Logan R, Biratu MA, Busila MA, Busto IF, Caldwell N, Chestnut P, et al., Waterman M. 2025. The Complete Genome Sequences of Bacteriophages ASegato, DejaVu, Judebell, and RicoCaldo isolated using Microbacterium foliorum. microPublication Biology. 10.17912/micropub.biology.001443

The authors correct the following:

One of the co-authors' names was misspelled; “Wesely Mills” has been corrected to “Wesley Mills.”

